# CC chemokine ligand 2 upregulates the current density and expression of TRPV1 channels and Na_v_1.8 sodium channels in dorsal root ganglion neurons

**DOI:** 10.1186/1742-2094-9-189

**Published:** 2012-08-08

**Authors:** Der-Jang Kao, Allen H Li, Jin-Chung Chen, Ro-Sun Luo, Ying-Ling Chen, Juu-Chin Lu, Hung-Li Wang

**Affiliations:** 1 Department of Physiology and Pharmacology, Chang Gung University School of Medicine, Kwei-San, 259 Wen-Hwa 1 road, Tao-Yuan 333, Taiwan; 2 Department of Anesthesiology, Chang Gung Memorial Hospital, Kwei-San, 5 Fu-Shing St, Tao-Yuan 333, Taiwan; 3 Department of Neurology, Chang Gung Memorial Hospital, Kwei-San, 5 Fu-Shing St, Tao-Yuan 333, Taiwan; 4 Chang Gung University of Science and Technology, Kwei-San, 261 Wen-Hwa 1 road, Tao-Yuan 333, Taiwan

**Keywords:** CC chemokine ligand 2, Dorsal root ganglion neurons, Transient receptor potential vanilloid receptor 1, Tetrodotoxin-resistant Na_v_1.8 sodium channel

## Abstract

**Background:**

Inflammation or nerve injury-induced upregulation and release of chemokine CC chemokine ligand 2 (CCL2) within the dorsal root ganglion (DRG) is believed to enhance the activity of DRG nociceptive neurons and cause hyperalgesia. Transient receptor potential vanilloid receptor 1 (TRPV1) and tetrodotoxin (TTX)-resistant Na_v_1.8 sodium channels play an essential role in regulating the excitability and pain transmission of DRG nociceptive neurons. We therefore tested the hypothesis that CCL2 causes peripheral sensitization of nociceptive DRG neurons by upregulating the function and expression of TRPV1 and Na_v_1.8 channels.

**Methods:**

DRG neuronal culture was prepared from 3-week-old Sprague–Dawley rats and incubated with various concentrations of CCL2 for 24 to 36 hours. Whole-cell voltage-clamp recordings were performed to record TRPV1 agonist capsaicin-evoked inward currents or TTX-insensitive Na^+^ currents from control or CCL2-treated small DRG sensory neurons. The CCL2 effect on the mRNA expression of TRPV1 or Na_v_1.8 was measured by real-time quantitative RT-PCR assay.

**Results:**

Pretreatment of CCL2 for 24 to 36 hours dose-dependently (EC_50_ value = 0.6 ± 0.05 nM) increased the density of capsaicin-induced currents in small putative DRG nociceptive neurons. TRPV1 mRNA expression was greatly upregulated in DRG neurons preincubated with 5 nM CCL2. Pretreating small DRG sensory neurons with CCL2 also increased the density of TTX-resistant Na^+^ currents with a concentration-dependent manner (EC_50_ value = 0.7 ± 0.06 nM). The Na_v_1.8 mRNA level was significantly increased in DRG neurons pretreated with CCL2. In contrast, CCL2 preincubation failed to affect the mRNA level of TTX-resistant Na_v_1.9. In the presence of the specific phosphatidylinositol-3 kinase (PI3K) inhibitor LY294002 or Akt inhibitor IV, CCL2 pretreatment failed to increase the current density of capsaicin-evoked inward currents or TTX-insensitive Na^+^ currents and the mRNA level of TRPV1 or Na_v_1.8.

**Conclusions:**

Our results showed that CCL2 increased the function and mRNA level of TRPV1 channels and Na_v_1.8 sodium channels in small DRG sensory neurons via activating the PI3K/Akt signaling pathway. These findings suggest that following tissue inflammation or peripheral nerve injury, upregulation and release of CCL2 within the DRG could facilitate pain transmission mediated by nociceptive DRG neurons and could induce hyperalgesia by upregulating the expression and function of TRPV1 and Na_v_1.8 channels in DRG nociceptive neurons.

## Background

Following tissue inflammation or peripheral nerve injury, several chemokines are released by invading immune cells or resident cells and believed to enhance the activity of nociceptive dorsal root ganglion (DRG) neurons, leading to hyperalgesia, allodynia and spontaneous pain [1-4]. Multiple lines of evidence suggest that chemokine CC chemokine ligand 2 (CCL2)/monocyte chemoattractant protein-1 plays an important role in mediating the peripheral sensitization of nociceptive DRG neurons and pain hypersensitivity of inflammatory or neuropathic pain [1,3,5]. Chemokine (C-C motif) receptor 2 (CCR2), the preferred receptor for CCL2, is expressed in DRG neurons, and CCL2 directly excites nociceptive DRG neurons [6,7]. Expression of CCL2 or CCR2 in neuronal and glial cells of the DRG has been shown to be upregulated in several animal models of inflammatory or neuropathic pain [8-14]. CCL2 expressed by DRG neurons is packaged into large dense core vesicles and released from activated DRG neurons [15]. Transgenic mice overexpressing CCL2 exhibited greater edema and augmented thermal hyperalgesia following tissue inflammation [16]. Tissue inflammation-induced or nerve injury-induced upregulation and release of CCL2 within the DRG could therefore enhance pain transmission mediated by nociceptive DRG neurons and induce hyperalgesia. The exact molecular mechanism by which CCL2 facilitates the nociceptive transmission of DRG nociceptive neurons is not completely understood.

Transient receptor potential vanilloid receptor 1 (TRPV1) is a nonselective cation channel mainly expressed in small-diameter and medium-diameter DRG sensory neurons and activated by capsaicin, noxious heat and low pH [17]. Activation of TRPV1 by noxious stimuli, which induces inward cationic currents and resulting action potentials in nociceptive DRG neurons, is responsible for conveying nociceptive information to spinal dorsal horn [18-20]. Under pathological conditions, TRPV1 expression in nociceptive DRG neurons is upregulated in the animal model of complete Freund’s adjuvant-induced inflammation or peripheral neuropathy [21-24]. Furthermore, TRPV1 antagonists including AS1928370 and SB-705498 also significantly reduce complete Freund’s adjuvant-induced or nerve injury-induced thermal hyperalgesia and mechanical allodynia [23,25,26]. Upregulated function of TRPV1 is therefore believed to mediate the sensitization of nociceptive DRG neurons and cause inflammatory or neuropathic hyperalgesia [4,18]. Interestingly, CCR2 – the CCL2 receptor – is found in TRPV1-expressing nociceptive DRG neurons [15]. A reasonable hypothesis is therefore that upregulated CCL2 induces pain hypersensitivity within the DRG by augmenting TRPV1 function in DRG neurons.

The tetrodotoxin (TTX)-resistant Na_v_1.8 sodium channel is almost exclusively expressed in small-diameter nociceptive neurons of the DRG [27-31] and plays an essential role in the upstroke of action potentials and continuous firing activity of DRG nociceptive neurons [29,32,33]. Accumulating data indicate that Na_v_1.8 expressed in nociceptive sensory neurons is not only involved in normal pain sensation but also plays an important role in inflammatory and neuropathic pain [29-31]. A decrease in behavioral responses to noxious thermal and mechanical stimulus as well as delayed inflammatory hyperalgesia were observed in Na_v_1.8 knockout mice [34]. Knockdown of Na_v_1.8 expression in the DRG by anti-sense oligodeoxynucleotides attenuated mechanical allodynia and thermal hyperalgesia caused by peripheral inflammation and nerve injury [35,36]. Peripheral inflammation or nerve injury has been shown to upregulate mRNA expression of Na_v_1.8 in nociceptive DRG neurons [37-39]. Furthermore, A-803467, a potent and selective Na_v_1.8 sodium channel blocker, inhibited nerve injury-induced mechanical allodynia and inflammation-induced thermal hyperalgesia [40]. Both CCR2 and Na_v_1.8 are found in small nociceptive DRG neurons [15,27,28]. CCL2 is therefore likely to cause pain hypersensitivity of nociceptive DRG neurons by upregulating the function of Na_v_1.8 sodium channels.

TRPV1 channels and TTX-resistant Na_v_1.8 channels are two major regulators of excitability and pain transmission in small-diameter nociceptive DRG neurons [18-20,29,30,33]. Following tissue inflammation or nerve injury, an elevated CCL2 level within the DRG could very probably cause peripheral sensitization of nociceptive DRG neurons and hyperalgesia by upregulating the function of TRPV1 channels and Na_v_1.8 sodium channels in DRG nociceptive neurons. In accordance with this hypothesis, the present study showed that CCL2 pretreatment significantly increased the current density of TRPV1 agonist capsaicin-evoked inward currents and TTX-resistant sodium currents in cultured small-diameter DRG neurons by upregulating mRNA expression of TRPV1 channels and Na_v_1.8 sodium channels, respectively.

## Methods

### Chemicals and reagents

DMEM/F12 and fetal bovine serum were purchased from GIBCO Life Technologies (Carlsbad, CA, USA). Recombinant rat CCL2 was obtained from R&D Systems (Minneapolis, MN, USA). Capsaicin, tetrodotoxin, CCR2 antagonist BMS CCR2 22, phosphatidylinositol-3 kinase (PI3K) inhibitor LY294002 and ERK 1/2 inhibitor U0126 were from Tocris Bioscience (Bristol, UK). Akt inhibitor IV was purchased from Calbiochem (Darmstadt, Germany). Trizol for RNA isolation and cDNA synthesis reagents were obtained from Invitrogen (Carlsbad, CA, USA). The SYBR Green PCR Master Mix kit was purchased from Applied Biosystems (Foster City, CA, USA).

### Primary neuronal culture of the dorsal root ganglion

Animals were handled according to protocols approved by the Animal Care and Use Committee of Chang Gung University. Three-week-old Sprague–Dawley rats were terminally anesthetized with sodium pentobarbital and were decapitated. Lumbar DRGs were dissected and incubated with DMEM/F12 containing collagenase type II (3 mg/ml; Sigma, St Louis, MO, USA) for 50 minutes at 37°C. After being washed, DRGs were further digested with trypsin (0.3 mg/ml; Sigma) dissolved in DMEM/F12 for 20 minutes at 37°C. Ganglia were then dispersed by a fire-polished Pasteur pipette, and dissociated cells were plated onto poly-l-ornithine-coated and collagen-coated dishes. DRG neurons were then cultured in DMEM/F12 supplemented with 10 % heat-inactivated bovine serum. Proliferation of non-neuronal cells was prevented by adding 10 μM 5′-fluoro-2′-deoxyuridine and 10 μM uridine into culture medium. Two-day-old or 3-day-old cultured DRG neurons were incubated with different concentrations of CCL2 (R&D Systems) for 24 to 36 hours in the presence of a cocktail of protease inhibitors (Sigma) and then used for electrophysiological recordings or RT-PCR assays. For control experiments, a cocktail of protease inhibitors (Sigma) was added to the culture medium.

### Whole-cell voltage-clamp recording of capsaicin-evoked currents or tetrodotoxin-resistant sodium currents

Small-diameter DRG neurons were voltage-clamped using the conventional whole-cell version of patch-clamp techniques. Patch pipettes with a resistance of 5 MΩ were fabricated from hard borosilicate glasses using a pipette puller (P-87; Sutter Instruments, Novato, CA, USA). For the recording of TRPV1 agonist capsaicin-evoked currents, the extracellular solution had the following composition: NaCl 145 mM, KCl 3 mM, CaCl_2_ 2 mM, MgCl_2_ 1 mM, glucose 12 mM, and HEPES 10 mM (pH 7.3 with NaOH). The patch electrode was filled with the following: KCl 35 mM, KF 100 mM, MgCl_2_ 1 mM, EGTA 5 mM, ATP 2 mM and HEPES 10 mM (pH 7.3 with KOH). A stock solution (5 mM) of capsaicin was made in ethanol and diluted in the external solution. Capsaicin was applied to DRG neurons using the fast perfusion SF77-B system (Warner Instruments, Hamden, CT, USA). To record TTX-resistant sodium currents, the external solution had the following composition: NaCl 140 mM, TEA-Cl 10 mM, CoCl_2_ 2 mM, MgCl_2_ 1 mM, glucose 12 mM, TTX 0.0005 mM and HEPES 10 mM (pH 7.3 with NaOH). The patch pipette was filled with the solution containing the following: CsCl 140 mM, MgCl_2_ 1 mM, EGTA 5 mM, ATP 2 mM and HEPES 10 mM (pH 7.2 with CsOH).

The membrane current or voltage recorded by the patch-clamp amplifier (Axopatch-200B; Axon Instruments, Sunnyvale, CA, USA) was filtered, digitized (Digidata 1200; Axon Instruments) and stored for later analysis. Liquid junction potentials were corrected, and the compensation circuitry of the amplifier was used to minimize the series resistance error. Holding potentials, data acquisition and data analysis were controlled by software pCLAMP 7.0 (Axon Instruments). The Prism program (GraphPad Software) was used to analyze the dose–response curve. Whole-cell patch-clamp recordings were performed at room temperature (24 to 25°C).

### Quantitative real-time RT-PCR assay

According to our previous study [41], Trizol reagent (Invitrogen) was used to prepare total RNA from cultured DRG neurons. Subsequently, the first-strand cDNA was synthesized in a reaction mixture containing total RNA (2 μg), 8 ng/μl oligodT primer, 1 mM each dNTP, 20 U ribonuclease inhibitor and 200 U SuperScript III RT (Invitrogen). The reaction was performed for 1 hour at 50°C and was terminated by incubating for 15 minutes at 70°C. Real-time PCR was carried out in the StepOne Real-Time PCR system (Applied Biosystems) using the SYBR Green PCR Master Mix (Applied Biosystems). The reaction mixture (40 μl) consisted of a cDNA aliquot, 400 nM forward or reverse primer and 1× SYBR Green PCR Master Mix containing AmpliTaq Gold DNA polymerase and SYBR Green 1 dye. The following primers were used for real-time PCR reactions: CCR2, 5′-GTTGGTGAGAAGTTCCGAAGGT-3′ and 5′-GGTCTGCTGTCTCCCTATAGAA-3′; TRPV1, 5′-TTTCAGGGTGGACGAGGTAAA-3′ and 5′-TGCCTGGGTCCTCGTTGA-3′; α subunit of Na_v_1.8, 5′-CCGGTGGAAGCAGGAAGA-3′ and 5′-AGGAGCGGTGCAGCATGTA-3′; and α subunit of Na_v_1.9, 5′-TGGACTTGCCCATGGTGAT-3′ and 5′-GGACCCTGGTAGTGAAAGCAAA-3′. PCR amplification was performed for 10 minutes at 95°C and was followed by 40 cycles of 15 seconds at 95°C and 1 minute at 60°C. PCR amplification of GAPDH mRNA was used as the normalization control. The relative change in mRNA expressions was determined by the equation:$Fold\;change=2^{-[\Delta \Delta Ct]},\Delta \Delta Ct={\left(Ct_{CCR2/TRPV1/Nav.1.8/Nav1.9}-Ct_{\mathrm{GAPDH}}\right)}_{CCL2}-{\left(Ct_{CCR2/TRPV1/Nav1.8/Nav1.9}-Ct_{\mathrm{GAPDH}}\right)}_{\mathrm{control}}$where Ct value is the cycle number at which the fluorescence signal crosses the threshold.

### Statistical analysis

Results are expressed as the mean ± standard error value of *n* experiments. Statistical significance among multiple experimental groups was determined by one-way analysis of variance followed by Dunnett’s test. An unpaired Student’s *t* test (two-tailed) was used to determine the significant difference between two groups of data. *P* <0.05 was considered significant.

## Results

### Chemokine CCL2 augments TRPV1 agonist capsaicin-evoked currents in small-diameter DRG neurons and upregulates mRNA expression of TRPV1 in cultured DRG neurons

**Figure 1:**
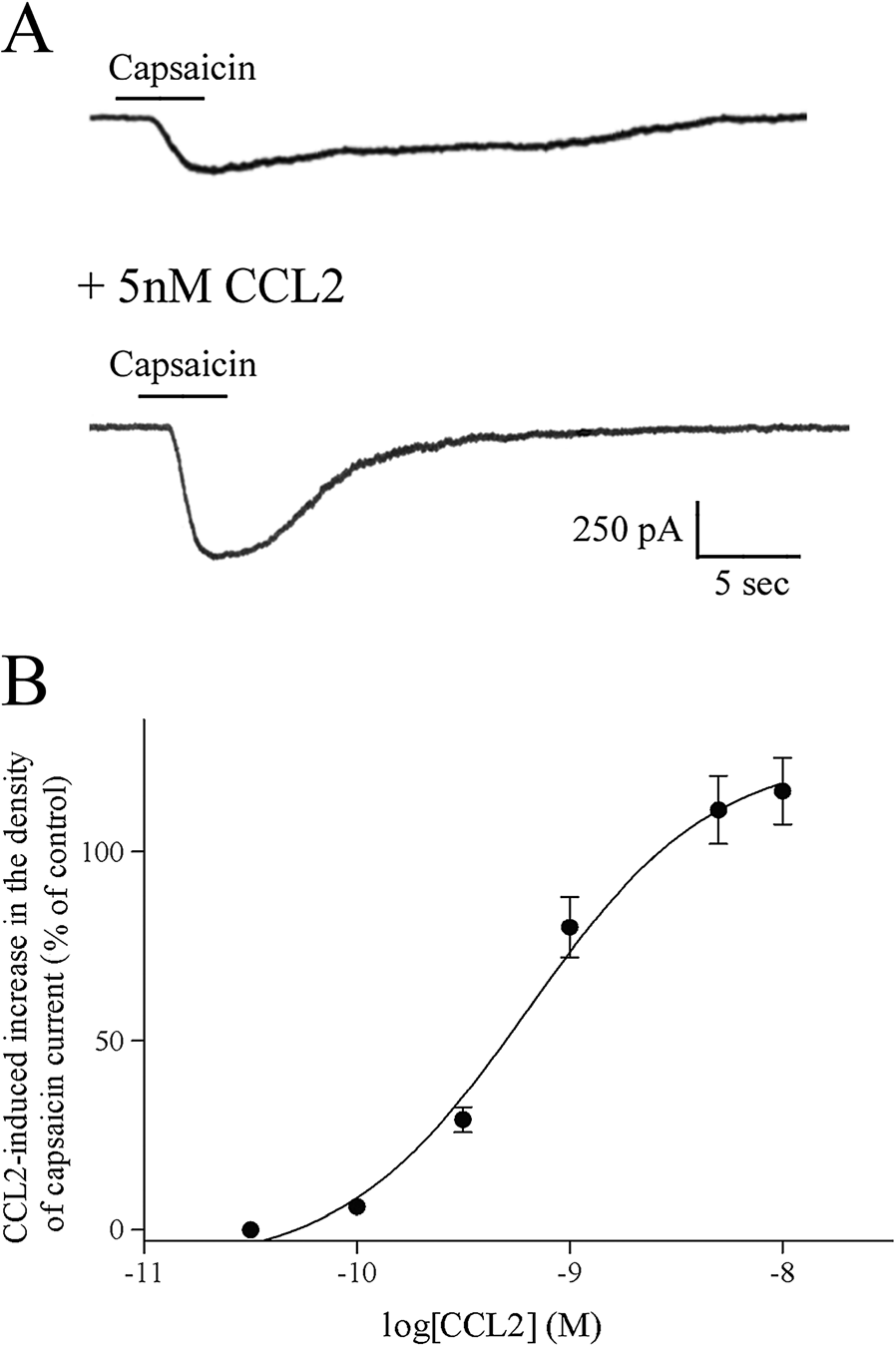
**CCL2 pretreatment augments capsaicin-evoked inward cationic currents in small-diameter dorsal root ganglion neurons.** (**A**) Compared with capsaicin (0.3 μM)-induced inward cationic current in a control small dorsal root ganglion (DRG) sensory neuron, the amplitude of capsaicin-evoked inward current was greatly increased in a small-diameter DRG neuron preincubated with 5 nM chemokine CC chemokine ligand 2 (CCL2) for 24 hours. Holding potential (V_H_) = −60 mV. (**B**) CCL2 pretreatment increased the density of capsaicin (0.3 μM)-induced inward currents with a concentration-dependent manner. Each point shows the mean ± standard error value of 10 neurons.

In the present study, we hypothesized that, during inflammatory or neuropathic pain, upregulated chemokine CCL2 induces hyperactivity of DRG nociceptive neurons and hyperalgesia by directly enhancing the function of TRPV1. To test this hypothesis, an *in vitro* inflammatory model of CCL2 upregulation in the DRG was prepared by pretreating primary culture of rat DRG neurons with different concentrations of CCL2 for 24 to 36 hours. According to a previous study [42], our *in vitro* inflammatory model was also believed to cause activity-dependent upregulation of CCR2 expression in DRG neurons. Consistent with this hypothesis, real-time RT-PCR assays demonstrated that pretreating cultured DRG neurons with 5 nM CCL2 for 24 to 36 hours induced a 3.2 ± 0.3-fold increase (*n* = 4 experiments) in the mRNA level of CCR2. Subsequently, TRPV1 agonist capsaicin-evoked inward cationic currents were recorded from control or CCL2-treated DRG sensory neurons (Figure 1).

According to the general belief that TRPV1 is mainly expressed in small-diameter DRG cells, which are believed to function as nociceptive neurons [19,20,43], cultured small DRG neurons (diameter = 15 to 20 μm) were selected for whole-cell patch-clamp recordings. The mean resting membrane potential, membrane capacitance and input resistance of control small-diameter DRG neurons were −59 ± 3 mV, 22 ± 3 pF and 520 ± 36 MΩ, respectively (*n* = 25). CCL2 (5 nM) pretreatment for 24 to 36 hours did not significantly affect the resting membrane potential (−55 ± 2 mV; *n* = 25), membrane capacitance (23 ± 3 pF) and input resistance (495 ± 25 MΩ) of small DRG sensory neurons. In accordance with previous studies [6,7], whole-cell current-clamp recording showed that application of 10 nM CCL2 induced a membrane depolarization (6 ± 1 mV; *n* = 8) from CCL2-pretreated cultured small DRG neurons.

**Figure 2:**
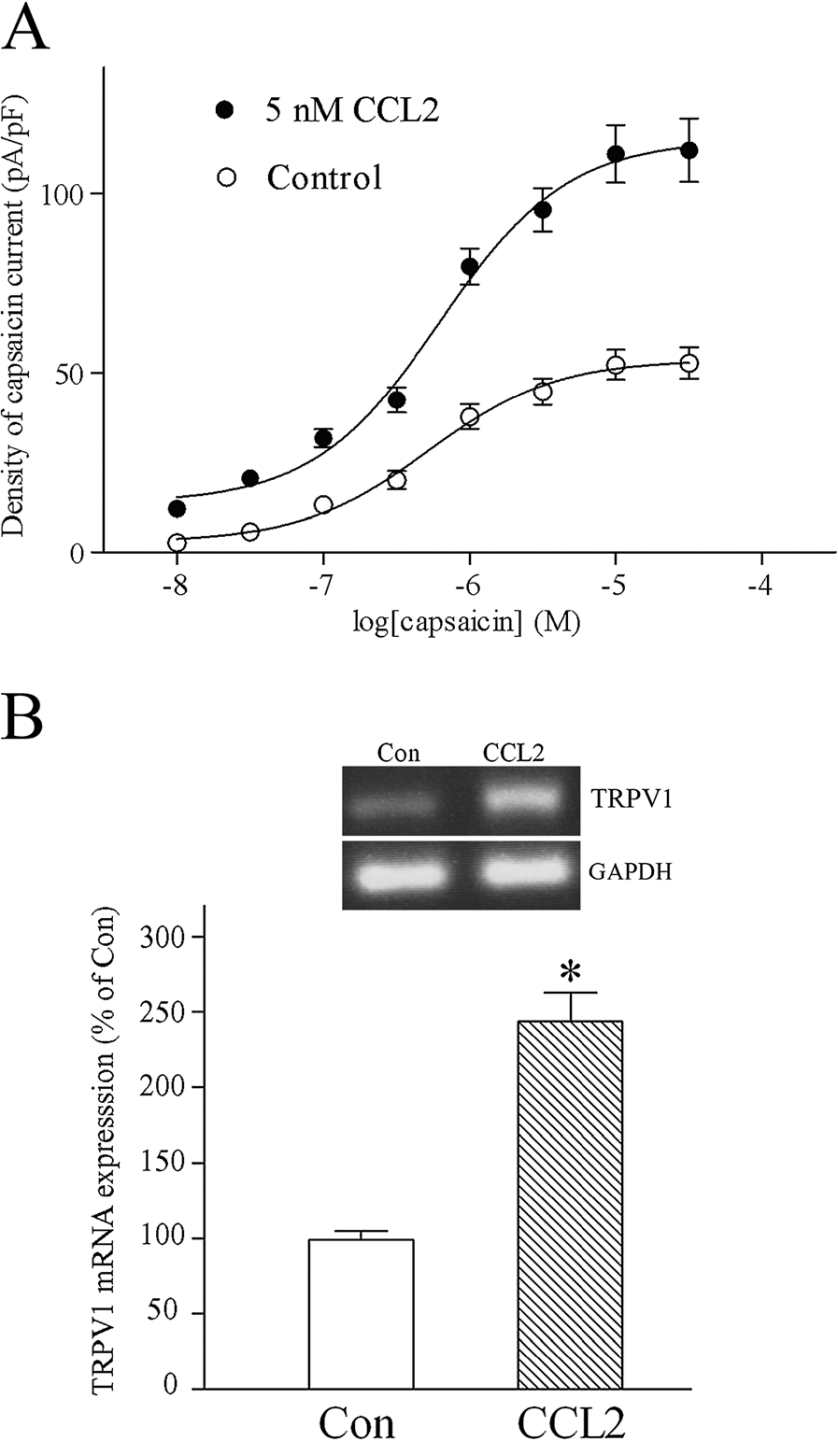
**CCL2 increases density of capsaicin-evoked inward currents by upregulating TRPV1 mRNA expression in sensory neurons.** (**A**) The concentration–response curve for the density of capsaicin-induced inward currents was obtained from small-diameter dorsal root ganglion (DRG) neurons in the absence and presence of chemokine CC chemokine ligand 2 (CCL2; 5 nM) pretreatment. Note that CCL2 preincubation increased the density of capsaicin currents without significantly affecting the EC_50_ value. Holding potential (V_H_) = −60 mV. Each point represents the mean ± standard error (SE) value of 12 neurons. (**B**) Quantitative RT-PCR assays showed that pretreating cultured DRG neurons with 5 nM CCL2 for 24 to 36 hours greatly increased the mRNA level of transient receptor potential vanilloid receptor 1 (TRPV1). Each bar shows the mean ± SE value of five experiments. *P <0.01.

Application of TRPV1 agonist capsaicin dose-dependently evoked inward cationic currents from small-diameter putative DRG nociceptive neurons at the holding potential of −60 mV (EC_50_ value = 0.5 ± 0.04 μM; Figures 1 and 2A). Following the preincubation of CCL2 (5 nM) for 24 to 36 hours, the mean amplitude of capsaicin (0.3 μM)-evoked cationic current was greatly increased in small-diameter DRG neurons (Figure 1A; control density of capsaicin current = 20 ± 2 pA/pF; with CCL2 pretreatment, density of capsaicin current = 42 ± 4 pA/pF; *n* = 12; holding potential = −60 mV). CCL2 pretreatment increased the density of capsaicin (0.3 μM)-induced current in a concentration-dependent manner (EC_50_ value = 0.6 ± 0.05 nM; Figure 1B). In the presence of the potent and specific CCR2 antagonist BMS CCR2 22 (0.5 μM) [44], CCL2 (5 nM) pretreatment failed to significantly increase the magnitude of capsaicin (0.3 μM)-evoked currents in small DRG sensory neurons (control density of capsaicin current = 21 ± 2 pA/pF; with CCL2 and BMS CCR2 22, density of capsaicin current = 23 ± 3 pA/pF; *n* = 5; holding potential = −60 mV).

Pretreating DRG neurons with 5 nM CCL2 for 24 to 36 hours increased the maximal magnitude of capsaicin-evoked inward currents without significantly affecting the EC_50_ value (control EC_50_ value = 0.5 ± 0.04 μM; with 5 nM CCL2 pretreatment, EC_50_ value = 0.6 ± 0.05 μM; Figure 2A). CCL2 is therefore not likely to augment capsaicin activation of TRPV1 by enhancing capsaicin affinity for TRPV1 channels. Instead, it is very likely that CCL2 increases the density of capsaicin-evoked inward currents by upregulating the expression level of TRPV1 in DRG sensory neurons. Consistent with this hypothesis, real-time RT-PCR assays demonstrated that, compared with control cultured DRG neurons, the mRNA level of TRPV1 was greatly increased in DRG sensory neurons pretreated with 5 nM CCL2 for 24 to 36 hours (Figure 2B). Our results strongly suggest that CCL2 augments TRPV1 function and enhances nociceptive transmission of small-diameter DRG neurons by upregulating TRPV1 mRNA expression.

### CCL2 augments capsaicin activation of TRPV1 and increases the TRPV1 mRNA level through activating the PI3K/Akt pathway

**Figure 3:**
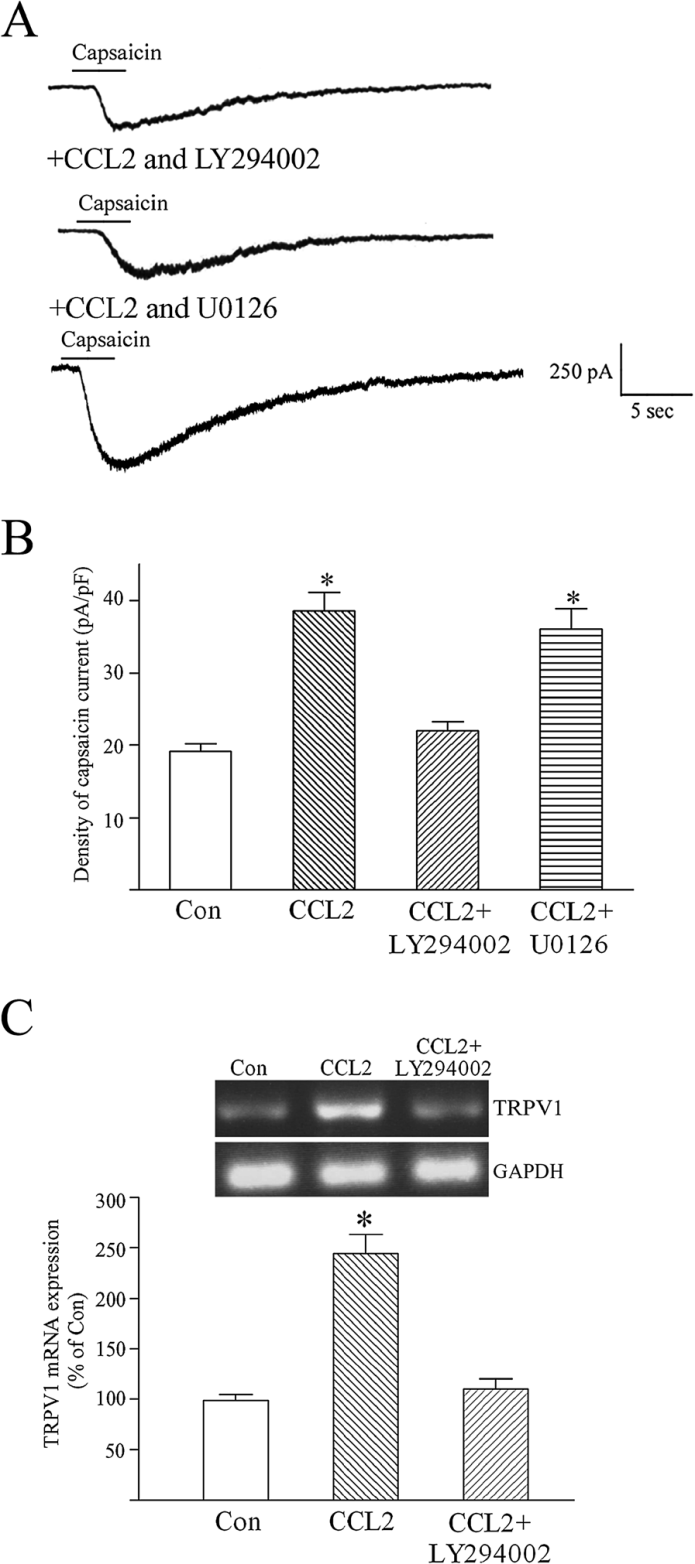
**CCL2 augments capsaicin-evoked inward currents and increases TRPV1 mRNA in neurons via activating phosphatidylinositol-3 kinase.** (**A**) Following co-treating cultured dorsal root ganglion (DRG) neurons with chemokine CC chemokine ligand 2 (CCL2) (5 nM) and phosphatidylinositol-3 kinase (PI3K) inhibitor LY294002 (10 μM) for 24 to 36 hours, CCL2 failed to significantly enhance the amplitude of capsaicin (0.3 μM)-evoked inward current in a small-diameter DRG neuron. In the presence of ERK 1/2 inhibitor U0126 (20 μM), CCL2 still greatly augmented the magnitude of capsaicin currents in a small DRG sensory neuron. Holding potential (V_H_) = −60 mV. (**B**) Pretreating small-diameter DRG neurons with 5 nM CCL2 significantly increased the density of capsaicin-evoked inward currents. LY294002 (10 μM) almost completely blocked CCL2 enhancement of capsaicin currents, and U0126 (20 μM) failed to affect CCL2 potentiation of capsaicin currents. Each bar shows the mean ± standard error (SE) value of 10 to 13 neurons. (**C**) RT-PCR assays showed that CCL2 (5 nM) pretreatment significantly increased the transient receptor potential vanilloid receptor 1 (TRPV1) mRNA level of cultured DRG neurons. In the presence of 10 μM LY294002, CCL2 pretreatment of DRG sensory neurons for 24 to 36 hours failed to upregulate TRPV1 mRNA expression. Each bar represents the mean ± SE value of five experiments. *P <0.01 compared with control neurons.

CCL2 activation of CCR2 has been shown to produce various cellular responses via two signal transduction pathways, the PI3K/Akt and ERK 1/2 cascades [45-48]. LY294002, a specific PI3K inhibitor [49,50], was used to test the involvement of PI3K in mediating CCL2 (5 nM) potentiation of capsaicin-evoked inward currents. In the presence of 10 μM LY294002, CCL2 pretreatment failed to increase the amplitude of capsaicin (0.3 μM)-evoked inward currents in small-diameter DRG neurons (Figure 3A,B). On the other hand, U0126 (20 μM) – a potent and specific inhibitor of ERK 1/2 – failed to affect CCL2 enhancement of capsaicin-induced inward currents in small DRG sensory neurons (Figure 3A,B). RT-PCR assays further demonstrated that LY294002 almost completely inhibited CCL2 upregulation of TRPV1 mRNA expression in DRG sensory neurons (Figure 3C).

**Figure 4:**
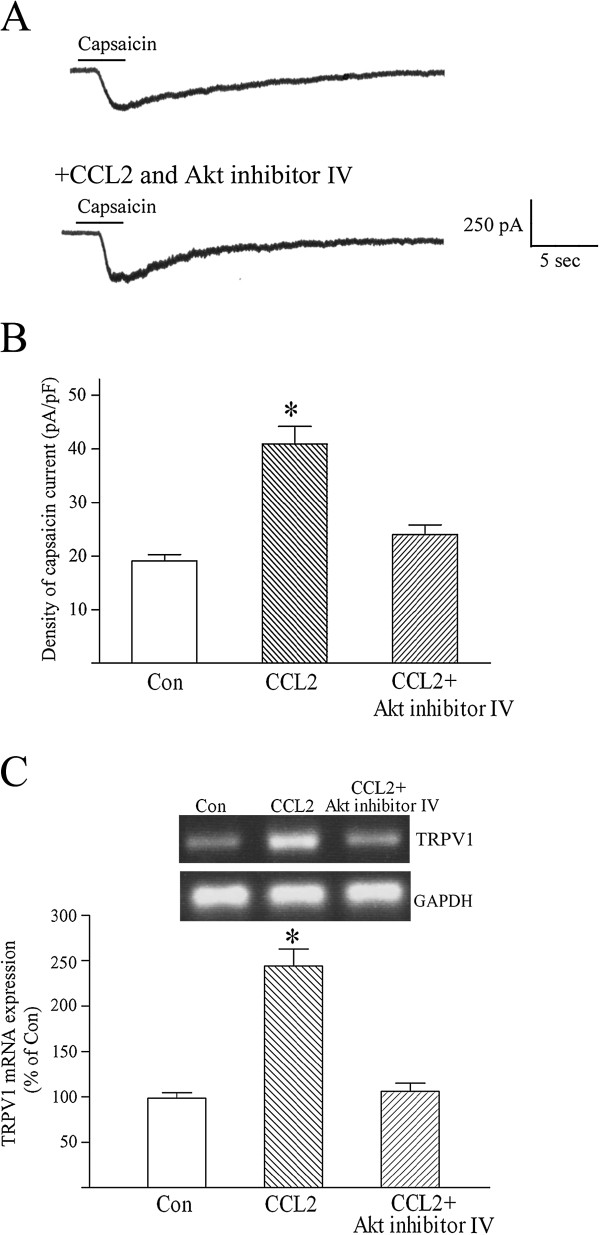
**CCL2 enhances capsaicin currents and upregulates TRPV1 mRNA expression in neurons via activating Akt.** (**A**) In the presence of Akt inhibitor IV (1 μM), chemokine CC chemokine ligand 2 (CCL2) (5 nM) pretreatment did not augment the magnitude of capsaicin (0.3 μM)-evoked inward current. Holding potential (V_H_) = −60 mV. (**B**) Akt inhibitor IV significantly blocked CCL2 upregulation of density of capsaicin currents. Each bar shows the mean ± standard error (SE) value of 10 neurons. (**C**) CCL2 pretreatment of dorsal root ganglion (DRG) neurons for 24 to 36 hours failed to increase transient receptor potential vanilloid receptor 1 (TRPV1) mRNA level in the presence of 1 μM Akt inhibitor IV. Each bar represents the mean ± SE value of five experiments. *P <0.01 compared with control neurons.

Akt/protein kinase B (PKB) is a critical downstream target of PI3K and mediates various PI3K-dependent signal pathways via phosphorylating target proteins [51,52]. The possible role of Akt/PKB in mediating CCL2 enhancement of capsaicin activation of TRPV1 cation channels was investigated using the Akt/PKB-specific inhibitor Akt inhibitor IV (1 μM) [49,50,53]. Pretreating cultured DRG neurons with CCL2 (5 nM) and Akt inhibitor IV for 24 to 36 hours did not significantly augment the magnitude of capsaicin-evoked inward currents (Figure 4A,B). RT-PCR assays also showed that Akt inhibitor IV completely blocked CCL2-induced upregulation of TRPV1 mRNA expression in cultured DRG neurons (Figure 4C). Our results suggest that CCL2 upregulates the expression and function of TRPV1 and facilitates nociceptive transmission of DRG sensory neurons by activating the PI3K/Akt signaling pathway.

### CCL2 increases the density of TTX-resistant sodium currents in small-diameter DRG neurons and the Na_v_1.8 mRNA level in cultured DRG neurons

**Figure 5:**
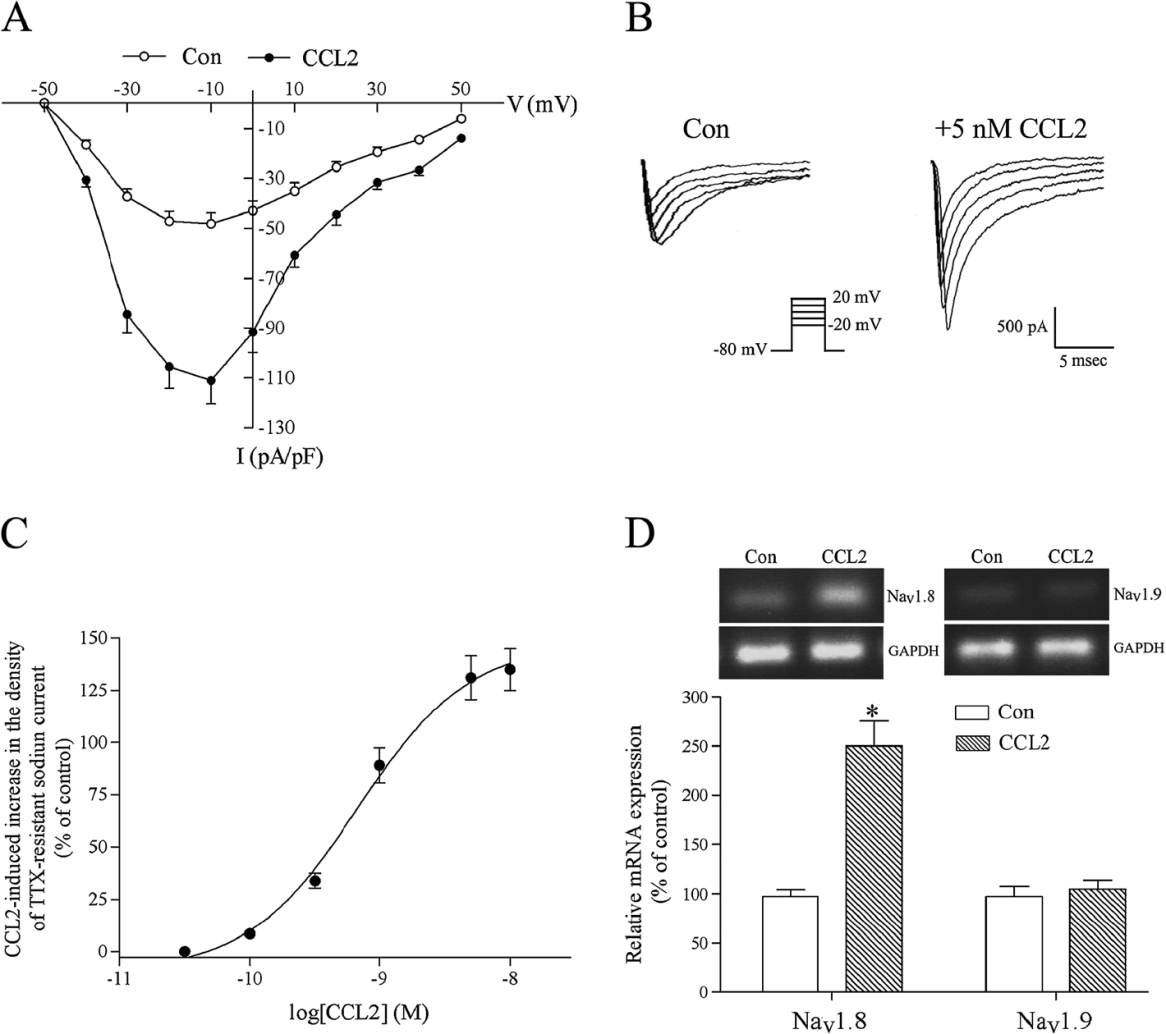
**CCL2 increases the density of tetrodotoxin-resistant sodium currents in small dorsal root ganglion sensory neurons.** (**A**) In the presence of 0.5 μM tetrodotoxin (TTX), the holding potential (V_H_) of small dorsal root ganglion (DRG) sensory neurons was held at −80 mV, and depolarizing steps (50 milliseconds) were applied from −50 mV to 50 mV with an increment of 10 mV. The I–V (current–voltage) curve of TTX-insensitive Na^+^ currents was then obtained from control or chemokine CC chemokine ligand 2 (CCL2; 5 nM)-pretreated small-diameter DRG neurons. Each point represents the mean ± standard error (SE) value of 10 neurons. (**B**) Traces of TTX-resistant sodium currents were evoked from a V_H_ of −80 mV to step potentials ranging from −20 mV to 20 mV. Compared with a control small DRG sensory neuron, the magnitude of TTX-insensitive Na^+^ currents was greatly increased in a CCL2-pretreated small-diameter DRG neuron. (**C**) CCL2 pretreatment increased the density of TTX-resistant sodium currents recorded at −10 mV in a dose-dependent manner. Each point shows the mean ± SE value of eight neurons. (**D**) Pretreating cultured DRG neurons with 5 nM CCL2 for 24 to 36 hours significantly upregulated mRNA expression of Na_v_1.8 without affecting the Na_v_1.9 mRNA level. Each bar represents the mean ± SE value of five experiments. *P <0.01 compared with control neurons.

To test the hypothesis that CCL2 enhances the excitability of DRG nociceptive neurons and causes hyperalgesia by augmenting TTX-resistant sodium currents, we recorded TTX-insensitive Na^+^ currents of small-diameter DRG neurons in the presence of 0.5 μM TTX. The membrane potential of small DRG sensory neurons was held at −80 mV, and depolarizing steps (50 milliseconds) from −50 mV to 50 mV were applied to cause the opening of TTX-resistant sodium channels (Figure 5A). In the present study, slowly inactivating TTX-insensitive Na^+^ currents of small-diameter DRG neurons displayed activation threshold of approximately −40 mV and peak amplitude at about −20 to −10 mV (Figure 5A,B). These electrophysiological properties are similar to those of TTX-resistant Na_v_1.8 sodium channels [27,29,34]. In addition to Na_v_1.8, Na_v_1.9 is another subtype of TTX-insensitive Na^+^ channels expressed in small-diameter DRG nociceptive neurons [29,31,54]. Na_v_1.8 current is believed to be the major TTX-resistant sodium current recorded from small DRG sensory neurons [29,54-56]. Previous studies also reported that Na_v_1.9 currents exhibited the phenomenon of washout and that the magnitude of Na_v_1.9 current is quite small with chloride-based internal solution used in the present study [29,55-57]. As a result, we failed to record TTX-insensitive Na_v_1.9 currents, which have a lower threshold of activation (−60 mV to −70 mV) and are persistent sodium currents [58,59], from cultured small DRG sensory neurons prepared in the present study (Figure 5). The TTX-resistant Na^+^ currents of small-diameter DRG neurons we recorded therefore predominantly result from the opening of Na_v_1.8 channels.

The current–voltage curves of TTX-insensitive sodium currents showed that, compared with control small DRG sensory neurons, the density of TTX-resistant Na^+^ currents at all step potentials was significantly increased in small-diameter DRG neurons pretreated with 5 nM CCL2 for 24 to 36 hours (Figure 5A,B). Pretreating small DRG sensory neurons with CCL2 increased the density of TTX-resistant sodium currents with a concentration-dependent manner (EC_50_ value = 0.7 ± 0.06 nM; Figure 5C). In the presence of the potent and specific CCR2 antagonist BMS CCR2 22 (0.5 μM) [44], 5 nM CCL2 pretreatment failed to significantly increase the density of TTX-insensitive Na^+^ currents in small DRG sensory neurons (control density of TTX-resistant Na^+^ currents at −10 mV = 47 ± 3 pA/pF; with CCL2 and BMS CCR2 22, density of TTX-insensitive Na^+^ currents = 51 ± 5 pA/pF; *n* = 5). CCL2 pretreatment did not significantly affect the threshold potential for activation, the step potential for peak value and the inactivation kinetics of Na_v_1.8-mediated TTX-insensitive sodium currents (Figure 5A,B). These results suggest that CCL2 increases the magnitude of TTX-insensitive Na^+^ currents by upregulating the expression of Na_v_1.8. In accordance with this hypothesis, real-time RT-PCR assays demonstrated that, compared with control DRG neurons, the mRNA level of Na_v_1.8 was significantly increased in DRG neurons pretreated with 5 nM CCL2 for 24 to 36 hours (Figure 5D). On the contrary, CCL2 pretreatment did not affect the mRNA level of TTX-resistant Na_v_1.9 in cultured DRG neurons (Figure 5D).

### CCL2 upregulates the density of TTX-resistant sodium currents and Na_v_1.8 mRNA expression by activating the PI3K/Akt pathway

**Figure 6:**
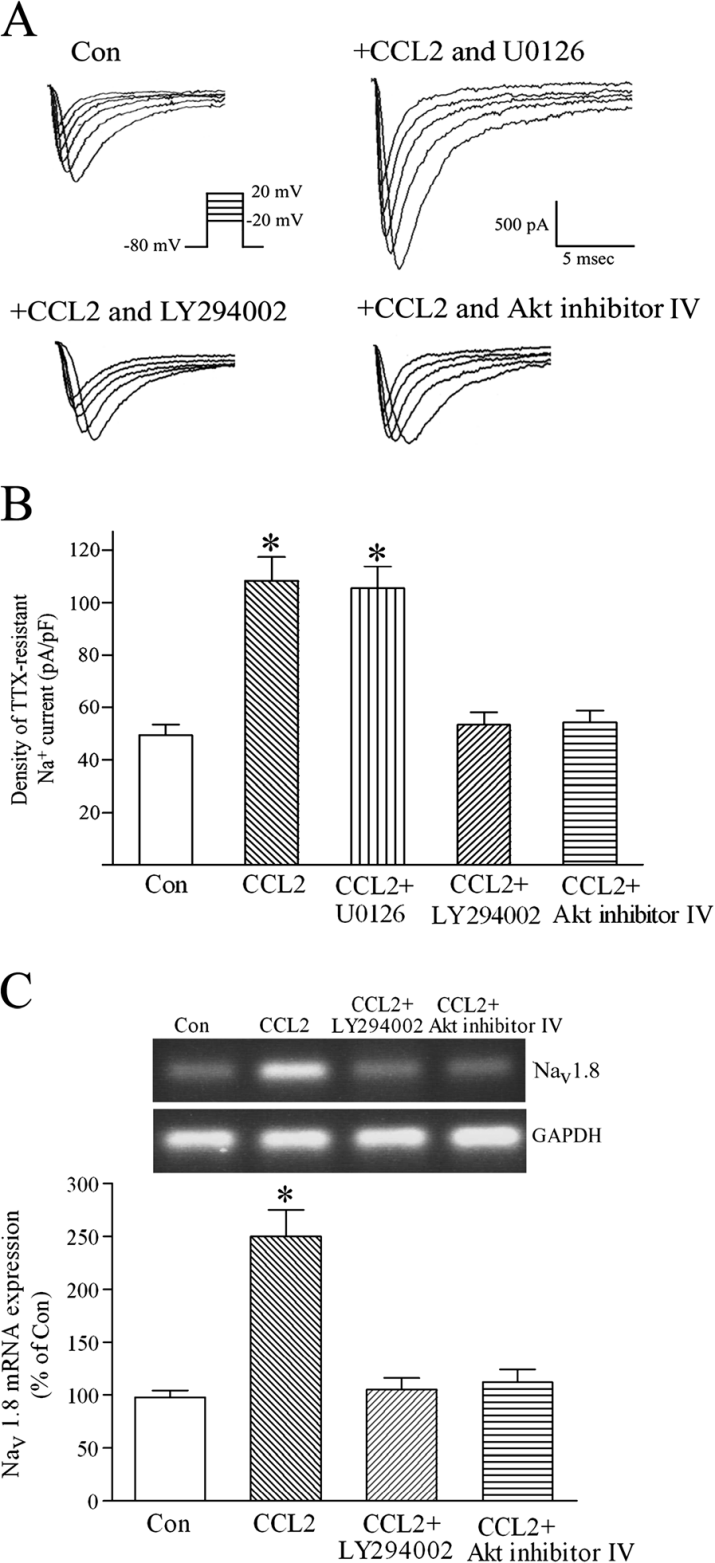
**CCL2 increases tetrodotoxin-resistant Na **^**+**^**current amplitude and Na**_**v**_**1.8 mRNA through activating the phosphatidylinositol-3 kinase/Akt pathway.** (**A**) Tetrodotoxin (TTX)-insensitive sodium currents were evoked from a holding potential (V_H_) of −80 mV to step potentials ranging from −20 mV to 20 mV. In the presence of ERK 1/2 inhibitor U0126 (20 μM), chemokine CC chemokine ligand 2 (CCL2) preincubation greatly increased the magnitude of TTX-resistant Na^+^ currents in a small dorsal root ganglion (DRG) sensory neuron. Pretreating small-diameter DRG neurons with 5 nM CCL2 did not augment the amplitude of TTX-resistant sodium currents in the presence of phosphatidylinositol-3 kinase (PI3K) inhibitor LY294002 (10 μM) or Akt inhibitor IV (1 μM). (**B**) Pretreating small-diameter DRG neurons with 5 nM CCL2 increased the density of TTX-insensitive sodium Na^+^ currents recorded at −20 mV. PI3K inhibitor LY294002 or Akt inhibitor IV almost completely inhibited CCL2 enhancement of TTX-resistant Na^+^ currents. Each bar shows the mean ± standard error (SE) value of 10 neurons. (**C**) Pretreating DRG neurons with 5 nM CCL2 for 24 to 36 hours significantly increased the mRNA level of Na_v_1.8. PI3K inhibitor LY294002 or Akt inhibitor IV significantly inhibited CCL2 upregulation of Na_v_1.8 mRNA expression. Each bar represents the mean ± SE value of five experiments. *P <0.01 compared with control neurons.

CCL2 probably increases the magnitude of TTX-insensitive Na^+^ currents and upregulates Na_v_1.8 mRNA expression via one of two CCL2-activated signaling pathways, the PI3K/Akt and ERK 1/2 cascades. In the presence of ERK 1/2 inhibitor U0126 (20 μM), CCL2 (5 nM) pretreatment still augmented the amplitude of TTX-resistant sodium currents in small DRG sensory neurons (Figure 6A,B). Co-treating cultured DRG neurons with CCL2 and PI3K inhibitor LY294002 (10 μM) almost completely blocked CCL2 enhancement of TTX-insensitive Na^+^ currents in small-diameter DRG neurons (Figure 6A,B). In the presence of Akt inhibitor IV (1 μM), CCL2 preincubation also failed to increase the magnitude of TTX-resistant sodium currents in small DRG sensory neurons (Figure 6A,B). Consistent with the results of whole-cell voltage-clamp recordings, real-time RT-PCR assays showed that co-treatment of LY294002 or Akt inhibitor IV greatly inhibited CCL2-induced upregulation of Na_v_1.8 mRNA expression in DRG sensory neurons (Figure 6C). These results suggest that CCL2 upregulates the expression and function of Na_v_1.8 channels and enhances the excitability of small DRG nociceptive neurons via the activating PI3K/Akt pathway.

## Discussion

Chronic inflammatory or neuropathic pain is associated with sensory disturbances characterized by hyperalgesia, allodynia and spontaneous pain [3,4,60]. Previous studies using animal models of inflammatory or neuropathic pain demonstrated that expression levels of CCL2 and its receptor CCR2 were upregulated in neuronal and glial cells of DRG [8-14]. Tissue inflammation-induced or nerve injury-induced upregulation and release of CCL2 within the DRG could therefore facilitate nociceptive processing by DRG sensory neurons and resulting hyperalgesia [1-3,5,16]. The exact molecular mechanism by which CCL2 enhances the excitability of DRG sensory neurons and induces the resulting pain hypersensitivity remains unknown.

Noxious stimuli-induced activation of TRPV1, a nonselective cation channel selectively expressed in nociceptive DRG neurons, induces inward cationic currents and action potentials, which then convey nociceptive information to spinal dorsal horn [18-20]. Peripheral inflammation or neuropathy upregulated TRPV1 expression in DRG nociceptive neurons [21-24], and knockdown of TRPV1 expression or TRPV1 antagonists significantly inhibited inflammation-induced or nerve injury-induced thermal hyperalgesia and mechanical allodynia [22,23,25,26]. CCR2 and TRPV1 are co-expressed in small DRG nociceptive neurons [15]. We therefore hypothesized that upregulated CCL2 facilitates nociceptive transmission of DRG sensory neurons by potentiating TRPV1 function in DRG sensory neurons. To test this hypothesis, we recorded TRPV1 agonist capsaicin-evoked inward cationic currents from control or CCL2-preincubated small putative DRG nociceptive neurons. CCL2 pretreatment dose-dependently upregulated the density of capsaicin-induced currents by increasing the mRNA level of TRPV1 in DRG sensory neurons. This finding strongly suggests that CCL2 enhances TRPV1 function and facilitates nociceptive transmission of small-diameter DRG nociceptive neurons by upregulating the expression of TRPV1. Interestingly, chemokine CCL3 is believed to cause inflammatory hyperalgesia by potentiating capsaicin-gated TRPV1 channel activity in DRG nociceptive neurons [61]. Cytokine TNFα has also been shown to augment capsaicin-evoked response in DRG or trigminal sensory neurons by upregulating TRPV1 expression [62,63]. Together with our results reported here, these results suggest that upregulated expression and function of TRPV1 is one of common pathogenic mechanisms by which proinflammatory cytokines or chemokines induce the peripheral sensitization of DRG nociceptive neurons and cause inflammatory or neuropathic hyperalgesia.

The expression of TTX-insensitive Na_v_1.8 is mainly restricted to small DRG nociceptive neurons [27-31], and opening of Na_v_1.8 channels contributes to the upstroke of action potential and continuous discharge of DRG nociceptive neurons [32,33]. Upregulated mRNA expression of Na_v_1.8 was observed in DRG sensory neurons following peripheral inflammation or nerve injury [37-39], and knockdown of Na_v_1.8 expression or Na_v_1.8 sodium channel blocker blocked nerve injury-induced or inflammation-induced hyperalgesia [35,36,40]. CCR2 and Na_v_1.8 are co-expressed in small nociceptive DRG neurons [15,28]. We therefore hypothesized that CCL2 causes hyperactivity of DRG nociceptive neurons and hyperalgesia by upregulating the function of Na_v_1.8 and enhancing the membrane excitability of DRG sensory neurons. Consistent with this hypothesis, pretreating small-diameter DRG nociceptive neurons with CCL2 significantly increased the current density of Na_v_1.8-mediated TTX-resistant Na^+^ currents without affecting the activation threshold or kinetic properties. Further real-time RT-PCR assays demonstrated that CCL2 significantly upregulated Na_v_1.8 mRNA expression in DRG sensory neurons. These results propose that following tissue inflammation or peripheral nerve injury, upregulation of expression and current density of Na_v_1.8 caused by CCL2 and other proinflammatory cytokines or chemokines augments the membrane excitability and induces ectopic discharges of DRG sensory neurons, leading to the development of inflammatory or neuropathic pain. In accordance with our hypothesis, chemokine CXCL1 has been shown to increase the current density of TTX-resistant currents and the mRNA level of Na_v_1.8 in small-diameter DRG sensory neurons [57]. Following the nerve injury, TNFα also increased the amplitude of TTX-insensitive sodium currents in DRG nociceptive neurons by upregulating mRNA expression of Na_v_1.8 [38,39].

Our results suggest that an elevated CCL2 level following tissue inflammation or nerve injury could cause peripheral sensitization of DRG nociceptive neurons and hyperalgesia by upregulating the expression and function of TRPV1 channels and Na_v_1.8 sodium channels. CCL2 has been shown to produce various cellular responses via two signal transduction pathways, the PI3K/Akt and ERK 1/2 cascades [45-48]. U0126, a potent and specific inhibitor of ERK 1/2, failed to block CCL2 enhancement of capsaicin-induced inward currents and TTX-resistant sodium currents in small DRG sensory neurons. In the presence of the specific PI3K inhibitor LY294002, CCL2 pretreatment failed to increase the current density of capsaicin-evoked inward currents or TTX-insensitive Na^+^ currents and the mRNA level of TRPV1 or Na_v_1.8. Specific Akt/PKB inhibitor IV also almost completely blocked CCL2-induced enhancement of capsaicin-evoked currents or TTX-resistant sodium currents and upregulation of TRPV1 or Na_v_1.8 mRNA expression. These results strongly suggest that CCL2 upregulates the expression and function of TRPV1 or Na_v_1.8 channels and enhances membrane excitability and nociceptive transmission of DRG sensory neurons via activating the PI3K/Akt signaling pathway. Consistent with our results, several lines of evidence suggested that activation of the PI3K/Akt pathway is involved in peripheral sensitization of DRG nociceptive neurons and subsequent development of inflammatory or neuropathic pain [64]. PI3K and Akt are expressed in small DRG nociceptive neurons, and the expression of active phospho-Akt in nociceptive DRG neurons is upregulated in the rat model of inflammatory or neuropathic pain [49,53,65]. Administration of PI3K inhibitor LY294002 and Akt inhibitor IV also inhibited capsaicin-induced or nerve growth factor-induced hyperalgesia and pain hypersensitivity observed in the animal model of neuropathic pain [49,53,66].

A previous study reported that brief pretreatment of monocyte chemoattractant protein-1/CCL2 caused sensitization of the capsaicin-evoked increase in the intracellular Ca^2+^ level in DRG sensory neurons [15]. Monocyte chemoattractant protein-1/CCL2-induced sensitization or transactivation of TRPV1 channels, which is mediated by phospholipase C and protein kinase C signaling pathways [15], could enhance the function of TRPV1 channels and cause pain hypersensitivity. During an *in vivo* pathological condition of tissue inflammation or peripheral nerve injury, the continuous presence of a high level of CCL2 should cause both upregulation of the TRPV1 mRNA level reported in the present study and transactivation of TRPV1 channels reported previously [15]. Following tissue inflammation or peripheral nerve injury, CCL2 therefore probably enhances the function of TRPV1 channels and facilitates the nociceptive transmission of DRG nociceptive neurons via two different molecular pathogenic mechanisms: PI3K/Akt-mediated upregulation of TRPV1 mRNA expression, and phospholipase C/protein kinase C-mediated transactivation of TRPV1 channels.

Akt mediates PI3K-dependent cellular responses via phosphorylating various target proteins including transcription factors [51,52]. The CCL2-activated PI3K/Akt pathway therefore probably increases the mRNA level of TRPV1 or Na_v_1.8 in DRG sensory neurons by enhancing the transcription of TRPV1 or the Na_v_1.8 gene. Functional promoter analysis indicated that the neuron-specific proximal promoter region of the Na_v_1.8 gene expressed in rat DRG neurons contained putative binding sites for transcription factor SP1 [67]. A recent chromatin immunoprecipitation analysis of DRG tissue demonstrated that the endogenous TRPV1 P2-promoter contains GC-box binding sites of transcription factor Sp1. Overexpression of Sp1 in cultured DRG neurons caused an increase in TRPV1 mRNA, and knockdown of Sp1 mRNA resulted in a decrease in TRPV1 mRNA [68]. Transcription factor SP1 therefore plays an important role in activating TRPV1 or Na_v_1.8 mRNA transcription in DRG sensory neurons. Interestingly, epidermal growth factor-induced activation of the PI3K/Akt pathway upregulated the mRNA expression of vascular endothelial growth factor in cell lines through Akt-mediated phosphorylation of transcription factor Sp1 and subsequent increased Sp1 binding to the vascular endothelial growth factor promoter [69]. Bcl-w-stimulated PI3K/Akt signaling also caused Sp1 activation and the resulting increased matrix metalloproteinase-2 expression in gastric adenocarcinoma cell lines [70]. CCL2 activation of Akt therefore possibly enhances the transcriptional activity of TRPV1 or the Na_v_1.8 gene and increases the mRNA level of TRPV1 or Na_v_1.8 by phosphorylating and activating transcription factor Sp1. Further research is required to investigate the exact molecular mechanism by which the CCL2-activated PI3K/Akt pathway upregulates mRNA expression of TRPV1 or Na_v_1.8 in DRG sensory neurons.

## Conclusions

In summary, our results demonstrate that CCL2 increased the density of TRPV1 agonist capsaicin-induced currents and Na_v_1.8-mediated TTX-resistant Na^+^ currents in small putative DRG nociceptive neurons. Further studies showed that CCL2 increased the function and mRNA level of TRPV1 channels and Na_v_1.8 sodium channels in DRG sensory neurons via activating the PI3K/Akt signaling pathway. These findings suggest that, following tissue inflammation or peripheral nerve injury, upregulation and release of CCL2 within the DRG could facilitate pain transmission mediated by nociceptive DRG neurons and could induce hyperalgesia by upregulating the expression and function of TRPV1 channels and Na_v_1.8 channels in DRG nociceptive neurons.

## Abbreviations

CCL2, CC chemokine ligand 2; CCR2, Chemokine (C-C motif) receptor 2; DMEM, Dulbecco's Modified Eagle Medium; DRG, Dorsal root ganglion; EC50, Half-maximal effective concentration; PCR, Polymerase chain reaction; PI3K, Phosphatidylinositol-3 kinase; PKB, Protein kinase B; RT, Reverse transcriptase; TNF, Tumor necrosis factor; TRPV1, Transient receptor potential vanilloid receptor 1; TTX, Tetrodotoxin.

## Competing interests

The authors declare that they have no competing interests.

## Authors’ contributions

H-LW, AHL, J-CC and R-SL designed the study. D-JK, AHL, Y-LC and J-CL performed the experiments. H-LW, D-JK, AHL, J-CC and R-SL discussed the results and prepared the manuscript. All authors read and approved the final version of this manuscript.
